# Tumor microenvironment and immunology of cholangiocarcinoma

**DOI:** 10.20517/2394-5079.2021.140

**Published:** 2022-03-10

**Authors:** Massimiliano Cadamuro, Luca Fabris, Xuchen Zhang, Mario Strazzabosco

**Affiliations:** 1 Department of Molecular Medicine (DMM), University of Padua, Padua 35131, Italy; 2 Department of Pathology, Yale School of Medicine, New Haven, CT 06510, USA; 3 Liver Center, Department of Internal Medicine, Yale School of Medicine, New Haven, CT 06520, USA

**Keywords:** Tumor reactive stroma, extracellular matrix, immunotherapy, checkpoint inhibitor, immune escape

## Abstract

Cholangiocarcinoma (CCA), an aggressive tumor originating from both intra- and extra-hepatic biliary cells, represents an unmet need in liver oncology, as treatment remains largely unsatisfactory. A typical feature of CCA is the presence of a complex tumor microenvironment (TME) composed of neoplastic cells, a rich inflammatory infiltrate, and cancer-associated fibroblasts and desmoplastic matrix that makes it extremely chemoresistant to traditional chemotherapeutic drugs. In this review, we describe the cell populations within the TME, in particular those involved in the innate and adaptive immune response and how they interact with tumor cells and with matrix proteins. The TME is crucial for CCA to mount an immune escape response and is the battlefield where molecularly targeted therapies and immune therapy, particularly in combination, may actually prove their therapeutic value.

## INTRODUCTION

Cholangiocarcinoma (CCA) is a highly malignant cancer that can develop from different segments of the biliary tree. The anatomical classification does not include gallbladder and ampullary cancers and distinguishes among intrahepatic (iCCA) that develops inside the liver up to the secondary bile ducts (< 10%), perilar (pCCA, also called Klastin’s tumor) that grows between the secondary biliary branches and the insertion of the cystic duct into the common bile duct (45%), and distal (dCCA) (45%) that is confined to the area between the origin of the cystic duct and the ampulla of Vater^[1,2]^. Despite recent progresses, the prognosis of CCAs has not substantially improved and five-year survival remains very low (5%-20%) with high post-surgical recurrence rates^[3]^.

CCA incidence shows a strong geographical variation, ranging from 0.4:100,000 inhabitants in Canada to 85:100,000 in the northeast of Thailand^[3]^. Furthermore, in the United States, distinct ethnic groups show different incidences with higher rates among Asians and Hispanics (2.8:100,000 and 3.3:100,000, respectively) and lower ones in Caucasians and African Americans (1.4:100,000 and 1.7:100,000)^[4]^. The most significant known risk factor for the development of CCA in East Asia is parasitic infestations of *Opisthorchis viverrini* or *Clonorchis sinensis*. After their encystation in the biliary network, these parasites cause chronic irritation, leading to neoplasm development^[5]^. In Western countries, the most prominent risk factor for CCA development is the primary sclerosing cholangitis (PSC) with an odds ratio (OR) of 164 (CI: 73.3-369, *P* < 0.001)^[6]^. PSC is an inflammatory disease affecting both the intra- and extra-hepatic biliary tract, causing inflammation of the biliary epithelium, periductal fibrosis, and biliary stenosis^[5]^. Other additional risk factors, particularly for iCCA, are HBV- and HCV-related cirrhosis, choledochal cysts, cholelithiasis, type 2 diabetes mellitus, obesity, non-alcoholic fatty liver disease, smoking, and hypertension^[7,8]^. It is worth noting that all these conditions are associated with liver inflammation.

Genetically, CCA is a heterogeneous tumor. CCAs originating from large ducts show a high mutation frequency of oncogenes and of tumor suppressor genes, such as Kirsten rat sarcoma virus (15%-30%) and tumor protein P53 (TP53) (10%-40%) They may also harbor mutations of BRAF, BRCA1 associated protein 1 (BAP1), phosphatidylinositol-4,5-bisphosphate 3-kinase catalytic subunit alpha (PIK3CA), guanine nucleotide binding protein GNAS, AT-rich interaction domain 1A (ARID1A), SMAD family member 4 (SMAD4), phosphatase and tensin homolog (PTEN), mouse double minute 2 homolog (MDM2), epidermal growth factor receptor (EGFR), and Erb-B2 receptor tyrosine kinase 2 (ERBB2), among others. Microsatellite instability is another prognostically and therapeutically relevant marker for CCA, as it has been shown that tumors with deficiency of mismatch DNA repair mechanisms (e.g., those associated with liver fluke infestation) are significantly more sensitive to immune checkpoint blockade^[9]^. In contrast, small duct type CCA exhibits a mass-forming growth pattern and exhibits isocitrate dehydrogenase 1/2 mutations (10%-30%) and fibroblast growth factor receptor 2 (FGFR2) fusions (10%-25%), among others^[10,11]^.

## REACTIVE TUMOR STROMA

**Figure 1.**
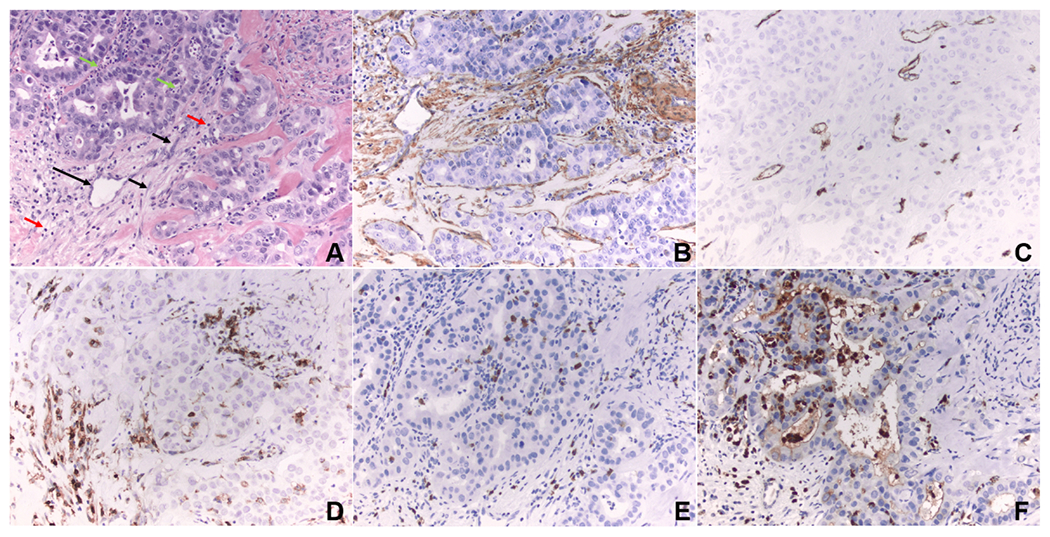
Tumor microenvironment. (A) Intrahepatic cholangiocarcinoma (hematoxylin and eosin stain, magnification 200×): black arrow, cancer-associated fibroblasts; green arrow, neutrophils; red arrow, tumor-infiltrating lymphocytes; long black arrow, microvessel. (B) Cancer-associated fibroblasts in the desmoplastic stroma are immunoreactive for α-smooth muscle actin, a biomarker of myofibroblast differentiation (immunohistochemistry, 200×). (C) Microvessels are highlighted by CD34 immunostain. (D) Tumor-infiltrating CD4 positive lymphocytes are highlighted by CD4 immunostain. (E) Tumor-infiltrating CD8 positive lymphocytes are highlighted by CD8 immunostain. (F) Tumor-associated neutrophils are highlighted by myeloperoxidase immunostain. (B-F) Brown stain indicates positive (mmunohistochemistry, magnification 200×).

**Figure 2.**
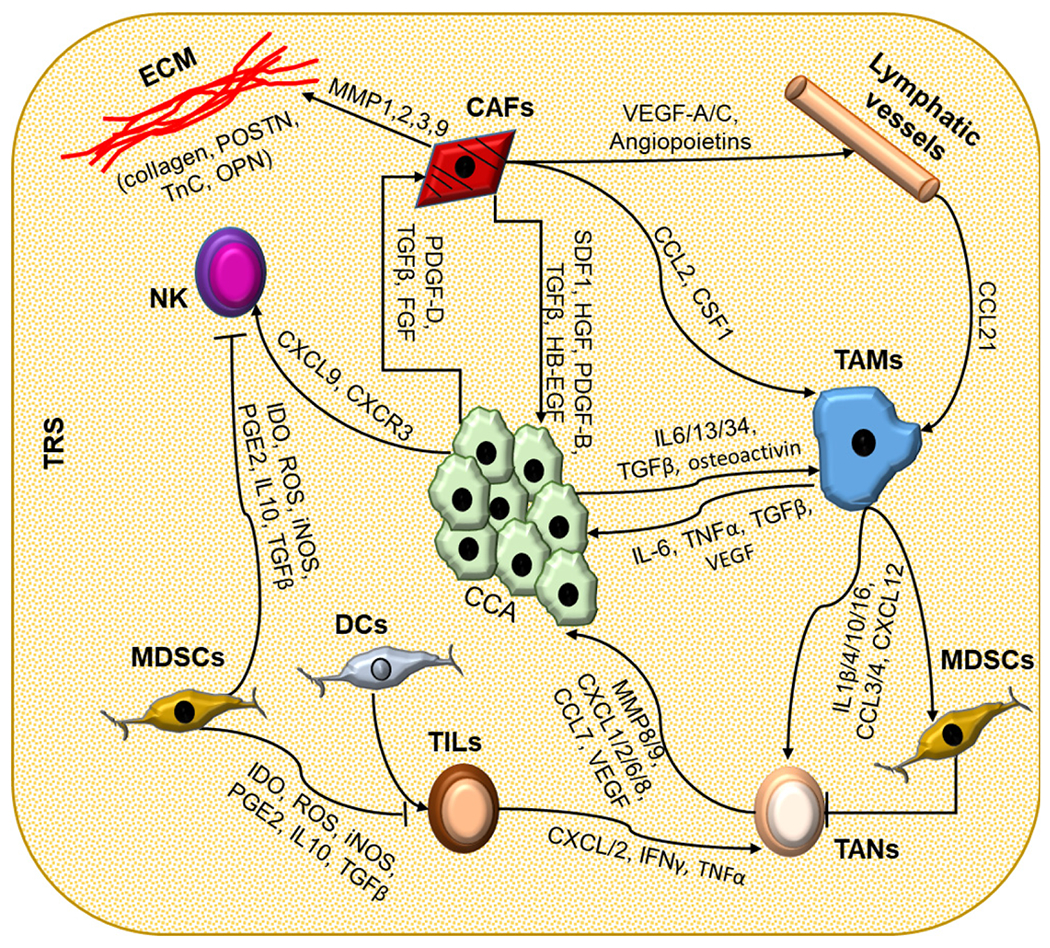
The complex interactions among the cell types composing TRS in CCA. In cholangiocarcinoma, neoplastic cells (CCA) are at the center of a complex interplay with a number of cell types that infiltrate the tumor microenvironment (TME) and a strongly modified extracellular matrix (ECM). Inflammatory cells and cancer-associated fibroblasts (CAFs), residing in close vicinity to CCA, can influence each other through the secretion of soluble mediators. CCA cells are able to recruit CAFs by secreting platelet-derived growth factor (PDGF)-D, transforming growth factor (TGF) β, and fibroblast growth factor (FGF); natural killer (NK) cells via the C-X-C motif ligand (CXCL) 9 - C-X-C motif chemokine receptor (CXCR) 3 axis; and cancer-associated macrophages (TAMs) through IL-6, IL-13, IL-34, TGFβ, and osteoactivin. In turn, these cells exert a trophic effect on neoplastic cells by secretion of soluble mediators. CAFs are also actively involved in the recruitment of lymphatic vessels and in the modifications of the ECM by secreting metalloproteinases (MMPs)-1, -2, -3, and -9, collagens, and other structural proteins such as osteopontin (OPN), tenascin C (TnC), and periostin (POSTN). The TME is also the site of an intense modulation of the innate and adaptive immune responses. Immune cells recruited into the tumor reactive stroma (TRS) influence each other in a difficult balance between immune surveillance and immune tolerance. Dendritic cells (DCs) stimulate the activation of tumor-infiltrating lymphocytes (TILs), while tumor-associated macrophages (TAMs) actively recruit tumor-associated neutrophils (TANs). Conversely, myeloid-derived suppressor cells (MDSC) inhibit the activity of immune cells, such as TILs, TANs, and NKs. The ability of each cancer to respond to immune therapy depends on the balance between these factors and the adaptation of the TME. Association of different targets (immune checkpoints and/or signaling targets) may be a pathway to therapeutic success.

Similar to other cancers, such as pancreatic or breast adenocarcinoma, CCA is characterized by an intense desmoplastic reaction [tumor reactive stroma (TRS)] supported by a rich cellular microenvironment and by modifications of the matrix composition. This tumor microenvironment (TME)^[2]^ has a structural component, the extracellular matrix (ECM), and a cellular component with a plethora of infiltrating cells. The matrix of the TME is significantly different from the normal one, in both quantity and quality. The TRS within the tumor is in fact continuously modified by the interaction between neoplastic and infiltrating cells. The cellular component of TME is variably composed of neoplastic epithelial cells, endothelial cells of the blood and lymphatic vessels, cancer-associated fibroblasts (CAFs), and cells of the innate [tumor-associated macrophages (TAM), tumor-associated neutrophils (TAN), dendritic cells (DC), natural killer (NK), and myeloid-derived suppressor cells (MDSC)] and adaptive immunity [tumor-infiltrating lymphocytes (TIL)]^[12,13]^ [Figure 1]. The structural component provides a dense and rigid scaffolding, which confers the characteristic desmoplasia to the tumor. This is composed of numerous and specific extracellular matrix proteins (see below). It is believed that the stroma does not have a simple passive function, but it actively participates in the intense communication between cells in the microenvironment and supports these interactions [Figure 2] and could be the target of therapeutic interventions [Table 1]^[2]^.

### Matrix

The matrix in the normal liver is usually limited to the portal space and the space of Disse. The persistence of a chronic inflammatory stimulus induces a process of pathologic repair that, losing the fine regulation and self-limitation, leads to scarring. During the process of cholangiocarcinogenesis, there is also an aberrant deposition of both structural and non-structural ECM components, which creates a thick and stiff layer of ECM proteins around the neoplastic bile ducts. Aberrant deposition of ECM components is considered a pathological hallmark of cholangiocarcinomas, and it is believed to be responsible for the pronounced aggressiveness of CCA and its low response to current therapies^[14,15]^. CCA cells secrete a wide range of proteolytic enzymes, such as metalloproteinase (MMP)-2 and -9, that dismantle the laminin-rich basal membrane, allowing the tumoral cells to invade the peritumoral matrix reaching lymphatic and blood vessels and thus to dive into the blood and lymphatic stream and disseminate to distant metastatic loci. This mechanism is further facilitated by the interaction of CCA cells with other cell types that that are recruited into the TME and primed to express a prosecretory phenotype able to further modify the surrounding ECM^[16,17]^. In CCA, there is indeed an abnormal deposition of several matricellular proteins, including periostin (POSTN), tenascin C (TnC), and osteopontin (OPN). These proteins are associated with an increase in tumor size and lymphatic metastasis and reduced overall survival^[3,12]^. These non-structural ECM proteins have an important role during embryonic development but in adult life are only expressed during tissue remodeling and wound repair^[18–20]^. POSTN can act as both a promoter and a suppressor of cancer cell invasiveness, interacting with other ECM proteins, such as collagen types I and V, fibronectin, TnC, and heparin, and contribute to the activation of pathways of tissue remodeling, fibrogenesis, cell motility, angiogenesis, tumor invasiveness, and metastasis. POSTN and TnC cooperate to promote metastasis through the activation of the Wnt and Notch signaling. POSTN is also capable of recruiting TAM, making this matrix protein a potential curative target for drug development^[21]^. TnC binds many ECM proteins, including fibronectin, POSTN, collagen, fibrillin-2, and proteoglycans, probably playing a structural role and defining the stiffness of the microenvironment. In iCCA, TnC is selectively expressed by the tumor invasion front, and its expression correlates with adverse outcomes^[22]^. OPN is a glycosylated phosphoprotein produced by many cell types that is normally involved in bone remodeling, immune-regulation, inflammation, and vascularization. The role of OPN in CCA has been covered recently^[23,24]^. OPN is in fact an important regulator of the repair response based on the progenitor liver cells that produce it and with an autocrine loop stimulates their proliferation and migration, which eventually leads to the ductular reaction. It also regulates the interaction between these cells and stromal cells; for example, through the mediation with transforming growth factor (TGF) β, it allows the activation of the fibroblasts that produce the other proteins, as well as the migration of macrophages^[23,24]^. For further information on the tumor matrix, see the work of Fabris *et al.*^[25]^.

### Cancer-associated fibroblasts

CAFs, the most represented cell type in the TME, are cells of mesenchymal origin that lay embedded into the tumoral ECM and have a prominent role in the production of ECM components and the degradation of the native ECM^[12,26]^. CAFs are constitutively activated fibroblasts and express α-smooth muscle actin (α-SMA), cluster of differentiation (CD) 10, and S100 calcium binding protein A4 (S100A4). The origin of these cells is debated, as it has been proposed that CAFs may derive from resident portal fibroblasts, from hepatic stellate, from bone marrow-derived mesenchymal cells, and/or epithelial/tumor cells, via EMT^[15]^ and are recruited around neoplastic biliary epithelia by the secretion of platelet derived growth factor (PDGF)-D^[27,28]^. Independently from the histogenesis, CAFs actively influence tumor progression thanks to a complex cross talk with the other components of the TME^[29]^. Once recruited, CAFs can stimulate tumor growth by secreting factors such as hepatocyte growth factor, TGFβ, PDGF-B, heparin-binding epidermal growth factor, and stromal cell-derived factor (SDF)-1^[12,30,31]^. Notably, SDF-1 is only weakly expressed by fibroblasts in the peritumoral area, but it is highly expressed and secreted by CAFs. Strong evidence for a role of CAFs in promoting CCA aggressiveness was demonstrated in a study in which a syngeneic rat model of CCA was treated with navitoclax (a small BH3-mimetic compound) to induce selective CAF depletion, thus suppressing tumor growth and improving host survival^[26]^. The relationship between CAF and other components of the TME, such as inflammatory cells and vessels, is very complex, and it is mediated by a series of growth factors [vascular endothelial growth factor (VEGF) and fibroblast growth factor (FGF)], TGFβ, cytokines, and chemokines [monocyte chemoattractant protein (MCP-1) and C-X-C motif ligand (CXCL) 12 and 14] and MMPs that promote tumor growth and spread, modifying the matrix, attracting the precursors of vascular and lymphatic vascular cells, and favoring an immunosuppressive microenvironment^[15]^.

### Endothelial cells

An important but still little studied element in the TME of CCA is its lymphatic vascular bed. Lymphatic metastatic spread occurs early during the course of CCA progression and often precludes curative surgical approaches. Notably, lymphatic endothelial cells are more represented in the TRS than blood endothelial cells and are localized in close proximity to CAFs^[15]^. Their involvement in the progression and metastatic spread of CCA seems to be due to the ability of both neoplastic and stromal cells to secrete lymphangiogenic growth factors (including VEGF-C, VEGF-D, and angiopoietins)^[32,33]^. Lymphatic vessels have large fenestrations that make them more permeable to the passage of immune cells and they secrete many chemokines [e.g., C-C motif ligand (CCL) 21] that promote the intravasation of macrophages and other inflammatory cells^[32,34]^. It has been shown that increased lymphatic density is associated with a worse prognosis and reduced disease-free and overall survival^[35,36]^.

### Innate immune cells

#### Tumor-associated macrophages

Two macrophage populations coexist in the TME of CCA: the liver resident macrophages (or Kupffer cells), and TAMs. On the contrary to the non-tumoral tissue, populated more by the M1 (or classically activated) subtype, which exert proinflammatory effects and defend the organism from invasion of pathogens, TAMs are mostly of the M2 (or alternatively activated) type and derive from circulating CD14^+^CD16^+^ monocytes that are usually involved in tissue repair and remodeling, angiogenesis, and matrix deposition^[37]^. TAMs play an active role in suppressing T cell activation and proliferation, in the promotion of angiogenesis, in the induction of tissue remodeling, and in stimulating apoptosis of M1 macrophages, which, however, contrast the neoplastic cells^[38,39]^. Whereas CAFs are spread over the entire tumor mass, TAMs are mainly located at the tumor invasive front, putatively recruited by neoplastic cells. CCA cells in fact secrete interleukins (IL) -6, -13, and -34, TGFβ, and osteoactivin, all molecules able to recruit monocytes and stimulate their M2 transdifferentiation^[40,41]^. Conversely, CAFs support TAM recruitment to the tumoral microenvironment by secreting CCL2 and colony stimulating factor 1 (CSF1)^[42]^. Many other secreted chemotactic mediators such as the cytokines IL-1β, IL-4, IL-10, and IL-16, CCL3, CCL4, and CXCL12 play a supportive role^[16,43]^.

TAMs can act as a trophic cell population for neoplastic cells by secreting IL6, tumor necrosis factor (TNF) α, TGFβ, and VEGF and activating cyclooxygenase-2 and WNT/β-catenin signaling^[44–47]^. TAM-secreted VEGF can also stimulate neoangiogenesis, thus contributing to CCA metastasis^[48]^. Moreover, TAMs are the main source of metalloproteases, in particular MMP-9, which, by degrading the matrix, favors metastasis^[39]^. Finally, TAMs can attract immunosuppressive cells, such as TANs and MDSCs, through the secretion of different soluble mediators (IL-4, IL-8, IL-10, CCL2, CCL17, and CCL22) to generate an immunosuppressive environment that favors the malignant behavior of CCA^[49]^.

#### Neutral killer cells

NK cells are a subpopulation of CD3^−^CD56^+^ lymphatic cells characterized by their ability to kill tumor- or virus-infected cells. Although NK cells are also known for their activity in recognizing and killing cancer cells, few studies have been performed in CCA. NKs can carry out their cytotoxicity through two pathways, one antigen-nonspecific, exploiting the release of enzymes such as perforin, proteases, and granzymes, and a direct one, through the activation of the Fas cell surface death receptor ligand (FasL)/TNF-related apoptosis-inducing ligand signal pathway^[50]^. The responsiveness of NK cells to the Fas/FasL pathway also has a drawback; in fact, a recent study demonstrated that, *in vitro*, iCCA cells express high levels of Fas and FasL, which induce apoptosis of NK cells, as an immune escape mechanism^[51]^. Conversely, the overexpression in CCA tumor cells of CXCL9, a ligand of C-X-C motif chemokine receptor (CXCR) 3, induces the recruitment of NK cells^[52]^ that infiltrate the tumor and positively correlated with postoperative overall survival in a cohort of 70 patients^[53]^. Similarly, using a xenograft model in which iCCA-derived HuCCT-1 cells were xenotransplanted into non-immunocompetent NCr athymic nude mice, infusion of NK cells (SMT01) induced significant inhibition of tumor growth^[54]^. Furthermore, *in vitro* treatment of HuCCT-1 and NK co-cultures with cetuximab, an EGFR inhibitor, demonstrated a significant increase in NK cytolytic activity against tumor cells^[55]^. These data suggest a potential use of NK in the treatment of CCA. NK cells are characterized by the expression of natural killer group 2 member D (NKG2D) receptor, a receptor whose polymorphisms are linked to the susceptibility to cancer development^[56]^. A study on 82 patients with eCCA who underwent surgical resection showed that overexpression of the NKG2D receptor on NK cells and its ligands in the cancer cells correlated with a better patient prognosis^[57]^.

#### Tumor-associated neutrophils

Despite their importance in the immune response^[58]^, there are very few studies on the involvement of neutrophils in the pathogenesis of CCA. Similar to TAMs, TANs are divided into two subcategories, N1, which is endowed with antitumor function, and N2, which has protumoral activity^[59]^. Circulating neutrophils are recruited to the tumor site by cells of the TME, such as CAFs [that secrete granulocyte-macrophage colony-stimulating factor (GM-CSF), granulocyte colony-stimulating factor (G-CSF), VEGF, and IL-1β], TAMs (IL-6 and IL-8), T lymphocytes (CXCL1, CXCL2, interferon γ, and TNFα)^[60]^. The neoplastic cells themselves secrete CXCL5, which induces neutrophils recruitment by activating the phosphatidylinositol 3-kinases/Akt and ERK1/2 pathways^[61]^. Once they reach the tumor site, TANs secrete a vast set of factors potentially involved in the biology of CCA, as mentioned above (such as MMP-8, MMP-9, CXCL1, CXCL2, CXCL6, CXCL8, CCL7, and VEGF)^[16,58]^. It has been shown that, in both iCCA and eCCA, the accumulation of TANs leads to a worse overall and disease-free survival of tumoral patients^[62–64]^. Unfortunately, only a few studies have evaluated the impact of TANs on CCA and they are mostly observational.

#### Dendritic cells

DCs are antigen-presenting cells and are usually found in small numbers in healthy tissues. It should be noted that the TME often has fewer DCs than the surrounding healthy tissue^[41]^. Similar to TANs, the studies regarding DCs in CCA are also rather sporadic. From a topographical point of view, mature DCs tend to accumulate on the tumor invasion front, while immature DCs are found in the tumor bulk^[65]^. A more recent study also demonstrated in a cohort of 350 patients with iCCAs that accumulation of DCs in the peritumoral tissue, but not in the CCA, is associated with a worse outcome^[66]^. A strong interaction between DCs and T cells has also been demonstrated; in fact, the inhibition of IL-10 and TGFβ receptors and the overexpression of cAMP-dependent protein kinase type I-alpha regulatory subunit (PRKAR1A) on DCs stimulates the antitumor activity of T cells against CCA^[67,68]^. Finally, a recent work with four different mouse models of iCCA showed that anti-CD40/PD-1 treatments, accompanied by gemcitabine/cisplatin, is able to activate the DC compartment and decrease tumor burden, an effect dramatically reduced by DC depletion^[69]^.

#### Myeloid-derived suppressor cells

A family of immune cells only recently studied in CCA is that of the MDSCs^[70]^. MDSCs are a large group of myeloid-derived cells whose number expands in diseases such as cancer or chronic inflammation and can exert an immunosuppressive effect^[70,71]^. MDSCs inhibit the action of cytotoxic T cells and NK cells by producing indoleamine 2,3-dioxygenase, reactive oxygen species (ROS), inducible nitric oxide synthase, prostaglandin E_2_, arginase, and immunomodulatory cytokines such as IL-10 and TGFβ^[70]^. A study performed on a very small cohort of 17 patients with CCA showed an increase in circulating MDSCs compared to controls^[72]^, an observation confirmed in a more recent study^[73]^. Zhang *et al.*^[74]^, using an Mdr2^−/−^ mouse in which iCCA was induced by hydrodynamic tail injection of plasmids known to favor its development (NICD + AKT and YAP + AKT), showed that accumulation of MDSCs favored tumor progression. Notably, gut sterilization was able to reduce MDSC recruitment. This observation demonstrates that hepatic recruitment of MDSCs can be modulated by the gut microbiota. Furthermore, using a different mouse model of iCCA [LSL-Kras^G12D^; Trp53^Flox/Flox^; Alb-Cre (KPPC) mouse], it was shown that neoplastic cells can recruit MDSCs via GM-CSF and that administering a blocking monoclonal antibody halts the recruitment of myeloid cells and decreases the growth and spread of the tumor^[73]^. In addition, the use of antibodies targeting a specific ApoE MDSC subset, coupled with TAM depletion, can increase the antitumoral effect of immune checkpoint blockade monotherapy^[75]^.

### Adaptive immunity

TILs are T cells that accumulate within the tumor stroma and counteract tumor development in an antigen-specific manner. The TIL population is composed by different cell types and includes CD4^+^ T cells (T helper or Th lymphocytes), CD4^+^CD25^+^ regulatory T cells (Tregs), CD8^+^ T cells (cytotoxic T lymphocytes), and CD20^+^ B lymphocytes. In general, in biliary tract tumors, the cells that mount the adaptive response tend to decrease during the process that goes from dysplasia to frank tumor and are also more numerous in eCCA than in iCCA^[76]^. The different components of TILs accumulate in specific areas of the tumor. While CD20^+^ B cells are present throughout the tumor, CD4^+^ cells accumulate in the peritumor area and CD8^+^ T cells on the tumor front^[62,76,77]^. Several studies showed that an enrichment in CD4^+^, CD8^+^, and CD20^+^ cells correlates with a better overall survival and lower recurrence rates in patients with both iCCA and eCCA^[63,76,78–81]^.

The adaptive immune response is also finely tuned by a set of stimulatory or inhibitory molecules expressed on the membrane of T cells called immune checkpoints that mostly function to avoid autoimmune reactions against *self*-cells. The downside of this mechanism is that it can be used by cancer cells to avoid being recognized by immune surveillance, a mechanism known as immune escape^[13,82]^. The main stimulatory molecules belonging to immune checkpoints include CD27, CD28, CD40, CD137, CD278, OX40, and glucocorticoid-induced TNF receptor, while among the inhibitory ones the best known and studied are cytotoxic T lymphocyte antigen-4 (CTLA-4), programmed death-1 (PD-1) and its ligand PD-L1, lymphocyte activation gene-3, T-cell immunoglobulin, and mucin protein-3^[83]^. Pharmacological blockage of inhibitory checkpoint molecules is currently exploited for the development of anti-tumor drugs, and there are several anti PD-1, PD-L1, and CTLA-4 molecules approved for use or currently in clinical trials for the treatment of several solid cancers, including CCA^[84–88]^. Data regarding the predictive value of PD-1 and PD-L1 expression on patient outcome are discordant and conflicting. One recent meta-analysis of 11 studies with more than 1000 patients showed that the expression of PD-L1 by tumor cells does not correlate with a worse overall survival of the patients even after stratifying by type of CCA^[89]^, but another meta-analysis provided opposite results^[90]^. As for PD-1, recent work has shown that, in iCCA, the increase in CD68^+^ macrophages and CD8^+^ T lymphocytes expressing PD-1 correlates with a worse postoperative survival^[91]^. Furthermore, in eCCA, high PD-1 expression appears to correlate with increased lymphatic metastases and lower patient survival^[92]^. Only one study evaluated the role of CTLA-4 expression as a prognostic indicator in CCA, demonstrating that, in eCCA, a high CTLA-4 H-score predicts better overall and disease-free survival^[93]^.

### Immunosuppressive tumor microenvironment

It is becoming clear that TME can generate an immunosuppressive environment and confer to the tumor cell a survival advantage by inducing tumor immune evasion. This is likely one of the main mechanisms responsible for the still disappointing results of immunotherapy in CCA^[3,12]^. For example, secretion of CCL2 by cells populating the TME, such as CAFs and tumor cells, leads to the enrichment of Tregs and of MDSCs. MDSCs in turn secrete ROS and other immunomodulatory compounds able to repress the activity of cytotoxic T cells (in particular CD8^+^ T cells) and NK cells. CAFs within the TRS of CCA secrete CXCL12, which may prevent migration of T cells^[94]^.

The effects of Tregs and their contribution to the pathogenesis of CCA, as well as their relevance as a prognostic index of CCA, are still debated. Data from two cohorts of patient with eCCA indicate the presence of Tregs as an indicator of poor outcome^[63]^, while other papers identify their over-representation as a positive prognostic factor^[76]^. From a biological point of view, Tregs secrete immunosuppressive mediators, such as IL10 and TGFβ, and further depress the antitumoral activity of CD8 T and NK cells^[95,96]^. A subset of Tregs, forkhead box P3 (FoxP3)^+^CD25^+^, bind IL-2, reducing the IL-2-mediated activation of the immune milieu^[95]^. The accumulation of FoxP3-positive Tregs is a distinctive trait of CCAs, showing worst outcome and greater tendency to lymphocyte metastasis^[63,66]^. Moreover, the accumulation of FoxP3^+^ Tregs is accompanied by an increase in expression of CTLA-4, also an indicator of poor outcome^[97]^.

## IMMUNOTHERAPEUTIC STRATEGIES TO TREAT CCA

To date, only pembrolizumab (an anti-PD-1 monoclonal antibody) is an approved treatment in CCA. Several other drugs and methods that exploit an immunotherapeutic approach are under evaluation. These studies are summarized in Table 2.

### Immune checkpoint inhibitors

Although the theoretical premises for the use of immune checkpoint inhibitors for the treatment of CCA are solid, the expectations were not fulfilled. The first clinical trials gave encouraging results, especially in patients with microsatellite instability due to mismatch repair deficiency. In these patients, the use of pembrolizumab, a humanized anti-PD-1 antibody (NCT01876511), gave a good response, with one patient in remission and three with stable disease^[98]^. Moreover, the analysis of five single-arm open-label clinical trials (KEYNOTE-012, -016, -028, -158, and -164) demonstrated an overall response rate (ORR) of 78%. However, these results were not confirmed in a larger study on 104 patients, KEYNOTE-158 (NCT02628067), in which the ORR was 5.8%. A clinical trial using nivolumab, an anti-PD-1 antibody (NCT02829918), failed to show favorable results^[99]^. CTLA-4 inhibitors are little studied in CCA and mostly in combined treatments in the hope to increase their efficacy. Some clinical trials have studied the combination of treatment with nivolumab and ipilimumab, an anti-CTLA-4 antibody (NCT02834013 and NCT02923934), in advanced solid tumors. Treatment of 39 patients showed an ORR of 23% and a disease control rate (DCR) of 44%, but with an overall survival (OS) of only 5.7 months^[100]^. The use of anti-CTLA-4 antibodies with anti-PD-1L antibodies is currently being evaluated in a phase II clinical trial (NCT04634058) whose results are not yet available. Pembrolizumab was also used in combination with levatinib, a tyrosine kinase inhibitor active on VEGFR1, VEGFR2, and VEGFR3, giving an ORR of 25% and a DCR of 78%^[101]^. Currently, a clinical trial for advanced CCA is in the recruitment phase (NCT04550624).

Another phase I study, KEYNOTE-098 (NCT02443324), used pembrolizumab in combination with ramucirumab, a monoclonal antibody against VEGFR2, but the response rate was 4% with disease stabilization in 35% of cases^[102]^. Tremelinumab, an anti CTLA-4 inhibitor, is being evaluated in a phase I clinical trial in combination with radiofrequency ablation (NCT01853618). Among 20 patients, 13% showed a partial response and 31% a stabilization of the disease. A recent study divided iCCA into four subtypes based on the differences of cell components of the TME (immune desert, immunogenic, myeloid, and mesenchymal). This stratification was proposed to better allocate patients to a more correct therapeutic intervention. In particular, the authors suggested treating with immune checkpoints inhibitor only the patients belonging to the immunogenic subtype, to maximize the potential anti-tumorigenic effects of the compounds^[103]^.

### Other immune strategies

These are mostly in the experimental or preclinical stage. The rationale behind the study of cancer vaccines is to identify proteins specifically expressed by cancer cells that could be recognized and destroyed by the immune cells. Few studies, usually composed of small cohorts, have been conducted in CCA. Identifying tumor-related proteins that can be specifically targeted by the vaccine is a daunting task. Studies conducted using Wilms’ tumor 1 (WT1) and Mucin-1 (MUC-1), proteins expressed by 70%-80% of iCCAs, as targets gave unsatisfactory results^[104,105]^. Similar disappointing results were obtained by co-treatment with gemcitabine and WT1 vaccine in four CCA patients^[106]^.

Another strategy to manipulate the immune system and train it to search and destroy tumoral cells is the use of adoptive cell therapies (ACTs), such as the autologous infusion of TILs or engineered T cells expressing with chimeric antigen receptor (CAR) or T cell receptors. The use of these therapeutic approaches in CCA are limited to case reports or small clinical trials, however the preliminary results are promising^[107–109]^. More recently, small phase I clinical trials have used CART technology. In one study, 14 patients with CCA were treated with infusions of EGFR-targeting CART cells (NCT01869166)^[110]^, while in a second clinical trial (NCT01935843) patients were infused with specific CARTs for human epidermal growth factor receptor 2^[111]^. Both trials resulted in an increase in disease-free survival with respect to patients treated with chemotherapy drug only. Several recent studies used new and improved (fourth-generation) CARTs that target proteins highly expressed by CCA, such as CD133^[112]^, MUC-1^[113]^, and integrin αVβ6^[114]^; these have been shown to be extremely efficient both in the expansion of the CART population and in the lysis of different CCA cell lines, *in vitro*.

## CONCLUSION

Despite the increased interest of the scientific community, treatment of cholangiocarcinomas remains an unmet need. Currently, a therapeutic option that has shown good efficacy is liver transplantation, which, only in very recent years, is being proposed for patients with iCCA and pCCA without distant metastases and with an early disease^[115]^. Aside from this option, there are no other truly effective treatment options. The genetic and phenotypic diversity that characterizes this family of rare cancers has negatively affected the progress in this field. An important strategy to overcome this impasse is to better understand the mechanisms that mediate the crosstalk between tumor cells, the variety of TME cells described above, and the components of the ECM. In recent years, several studies have aimed at classifying CCA on the basis of not only of their immunohistochemical and anatomopathological phenotype but also their genetics, signal pathways, and actionable targets. A seminal work by Sia *et al.*^[116]^ classifies iCCAs into proliferation class and inflammation class, based on specific signaling pathways and mutations. Notably, proliferation class shows a worse outcome with respect to the inflammatory one. Using a similar approach, mixed hepatocellular CCA (HCC/CCA)^[117]^ and eCCA^[118]^ have also been subcategorized. Using a molecular approach, eCCAs were classified in metabolic, mesenchymal, proliferative, and immune classes, all characterized in terms of mutations and modulation of different actionable targets. Furthermore, the development of single-cell RNA sequencing now allows analysis of the discrete cell populations in the TRS, including CAFs^[119]^ and immune response cells^[120]^, and to study in more detail the crosstalk between these cell milieus^[121]^. Finally, a recent study used nano liquid chromatography coupled to matrix-assisted laser desorption/ionization-time of flight (MALDI-TOF/TOF) analysis to study the composition of the matrix (or matrisome) in CCA^[122]^. Using this approach, these authors demonstrated that the aberrant deposition of collagen type III alpha 1 chain directly stimulates the migration of neoplastic cells^[122]^. Such studies will not only lead to a better understanding of the mechanisms of development and growth of the CCA but also open new avenues for a better allocation of patients to the most appropriate treatments. For example, when planning combination treatment with immune checkpoint inhibitors and other molecularly targeted drugs, a personalized approach based on genetic mutations and signaling pathways deregulated in specific CCA subclasses may confer a therapeutic advantage.

## Figures and Tables

**Table 1.** Clinical trials targeting ligands and receptors shred by different cell type of the TME

Ligands/receptors	Drugs	Clinical trial
VEGFRs, PDGFRs, c-MET	Lenvatinib	NCT03895970 NCT04211168 NCT04550624
	TT-00420	NCT04742959
	Pazopanib	NCT01855724
	Bevacizumab	NCT00350753 NCT00426829 NCT00356889 NCT01007552 NCT00410956 NCT04164069
	Apatinib	NCT03251443 NCT04454905
	Tivozanib	NCT04645160 NCT05000294
FGFRs	Pemigatinib	NCT02924376 NCT03656536
	Erdafitinib	NCT02699606
	E7090	NCT04238715
	Futibatinib	NCT02052778
	BGJ398	NCT03773302 NCT02150967
	RLY-4008	NCT04526106
	Derazantinib	NCT01752920
	INCB062079	NCT03144661
EGFR	A166	NCT03602079
	Varlitinib	NCT02609958
	Regorafenib	NCT02053376
	KSP/QRH dimer	NCT04304781
	Erlotinib	NCT00955149
	Panitumumab	NCT00397384 NCT00033462 NCT01320254
TGFβ	M7824	NCT04708067 NCT03833661 NCT04066491
CSF1	SNDX-6352	NCT04301778

VEGFR: Vascular endothelial growth factor receptor; PDGFR: platelet-derived growth factor receptor; FGFR: fibroblast growth factor receptor; EGFR: epidermal growth factor receptor; TGFβ: Transforming growth factor β; CSF1: colony stimulating factor 1.

**Table 2.** Clinical trials involving use of immune checkpoint inhibitors

Clinical trial	Phase	Drugs	Target	Status	Results
NCT01876511	2	Pembrolizumab	PD-1	Completed	1 patient in remission, 3 patients stable
NCT02628067	2	Pembrolizumab	PD-1	Recruiting	ORR: 5.8%
NCT02829918	2	Nivolumab	PD-1	Active, not recruiting	Failed
NCT02834013	2	Nivolumab Ipilimumab	PD-1 CTLA-4	Recruiting	ORR: 18% PFS: 2 months OS: 12 months
NCT02923934	2	Nivolumab Ipilimumab	PD-1 CTLA-4	Active, not recruiting	ORR: 23% OS: 5.7 months
NCT04634058	2	anti-CTLA-4 abs anti-PD-1L abs	CTLA-4 PD-1L	Not yet recruiting	No data available
NCT04550624	2	Pembrolizumab Lenvatinib Mesylate	PD-1 VEGFR1/2/3	Recruiting	ORR: 25% DCR: 78%
NCT04550624	2	Pembrolizumab Lenvatinib Mesylate	PD-1 VEGFR1/2/3	Recruiting	No data available
NCT02443324	1a/1b	Pembrolizumab Ramucirumab	PD-1 VEGFR2	Active, not recruiting	ORR: 4% Disease stabilization: 35%
NCT01853618	1/2	Tremelimumab TACE	CTLA-4	Completed	Partial response: 13% Disease stabilization: 31%
NCT01869166	1/2	CART-EGFR	EGFR	Unknown	Partial response: 29% Disease stabilization: 57%
NCT01935843	1/2	CART-HER-2	HER-2	Unknown	Partial response: 1 patients Disease stabilization: 5 patients

ORR: Overall response rate; PFS: progression-free survival; OS: overall survival; DCR: disease control rate.

## Data Availability

Not applicable.
